# Biomolecular evidence reveals mares and long-distance imported horses sacrificed by the last pagans in temperate Europe

**DOI:** 10.1126/sciadv.ado3529

**Published:** 2024-05-17

**Authors:** Katherine M. French, Adrianna D. Musiał, Maciej Karczewski, Linas Daugnora, Roman Shiroukhov, Katarzyna Ropka-Molik, Tadeusz Baranowski, Mindaugas Bertašius, Konstantin Skvortsov, Paweł Szymański, Izabela Mellin-Wyczółkowska, Anna Gręzak, Dariusz Wyczółkowski, Aleksander Pluskowski, Morten Andersen, Marc-Alban Millet, Edward Inglis, Richard Madgwick

**Affiliations:** ^1^ School of History, Archaeology and Religion, Cardiff University, Cardiff, UK.; ^2^ National Research Institute of Animal Production, Balice, Poland.; ^3^ Department of International Relations, University of Białystok, Białystok, Poland.; ^4^ Institute of the Baltic Region History and Archaeology, Klaipėda, Lithuania.; ^5^ Center for Baltic and Scandinavian Archaeology, Schleswig, Germany.; ^6^ Institute of Archaeology and Ethnology, Polish Academy of Sciences, Warsaw, Poland.; ^7^ Kaunas University of Technology, Kaunas, Lithuania.; ^8^ Institute of Archaeology, Russian Academy of Sciences, Moscow, Russia.; ^9^ Department of Archaeology, University of Warsaw, Warsaw, Poland.; ^10^ Masurian Archaeological Laboratory Rudka, Kętrzyn, Poland.; ^11^ Department of Archaeology, University of Reading, Reading, UK.; ^12^ School of Earth and Environmental Sciences, Cardiff University, Cardiff, UK.

## Abstract

Horse sacrifice and deposition are enigmatic features of funerary rituals identified across prehistoric Europe that persisted in the eastern Baltic. Genetic and isotopic analysis of horses in Balt cemeteries [1st to 13th centuries CE (Common Era)] dismantle prevailing narratives that locally procured stallions were exclusively selected. Strontium isotope analysis provides direct evidence for long-distance (~300 to 1500 kilometers) maritime transport of Fennoscandian horses to the eastern Baltic in the Late Viking Age (11th to 13th centuries CE). Genetic analysis proves that horses of both sexes were sacrificed with 34% identified as mares. Results transform the understanding of selection criteria, disprove sex-based selection, and elevate prestige value as a more crucial factor. These findings also provide evidence that the continued interaction between pagans and their newly Christianized neighbors sustained the performance of funerary horse sacrifice until the medieval transition. We also present a reference ^87^Sr/^86^Sr isoscape for the southeastern Baltic, releasing the potential of future mobility studies in the region.

## Teaser

Evidence from later Viking Age horse sacrifices shows maritime horse exchange connected Baltic pagan and Christian communities.

## INTRODUCTION

The public sacrifice and deposition of horses in human cemeteries are indelible features of Balt archeology from the 1st to 13th centuries CE and attest to the persistence of pagan belief systems (*1*). Research from the past 150 years documents how common, yet still noteworthy, these deposits remain (*2*–*6*). They provide a robust and well-preserved source of information on pre-Christian Balt ritualism and the socioeconomics of horsemanship (*7*–*14*). Horse sacrifices were highly visible public rites (*15*) requiring substantial investment of resources and revealing the cemetery as a vital node in Baltic sacred landscapes (*16*).

Sacrificial horse deposits are difficult to characterize because there is more variety than consistency in form and composition (*17*). Offering pits might include multiple horses, single complete horses, or partial animals. Whole horses are documented in different positions, most commonly crouched or laid out on one side (*9*, *18*, *19*). The deposition of partial animals would have been a bloody, macabre public spectacle, often involving decapitation, flaying, and halving or quartering of horses, or burying them alive (*8*, *9*). In many cemeteries, horses were buried separately from humans (*9*, *18*, *20*), whereas there are many examples of horses with overlain human cremations, known as double-layer cremations or *Aschenplätze* (*11*, *21*, *22*). Horses can be buried with (*23*–*25*) or without (*9*, *26*) riding equipment. Interment may have involved ancillary rituals with evidence for grave-side hearths (*20*) and the deposition of charred plants or charcoal from funerary pyres (*27*).

Despite this variety, two characteristics appeared to be consistent across time and space: the sex of the horse and the use of local breeds. Prior research indicates that sacrificed horses in cemeteries were all male, typically determined by the presence of canine teeth (*28*) and more rarely on pelvic morphology (*29*). The non-cemetery site of Poganowo IV [10th to early 12th centuries CE (Common Era)] is the sole contemporary Balt site with evidence for horse sacrifices with an equal proportion of male and female horses represented (*30*). This stark contrast suggests that the choice of male horses was vital to funerary contexts. Evocative narratives of horsemen and elite warrior funerals have been constructed, based on the consistent identification of male horses, the association of some horse sacrifices with male graves, and surviving textual evidence representing the sex of the stallion as a key element of the public performance (*8*, *11*, *12*, *31*, *32*). Recent reappraisals highlighting evidence for female graves associated with horse burials challenge these traditional narratives of horsemen burials but have not included a reanalysis of the sex of the horses themselves (*17*).

Biomolecular analysis can test the prevailing narrative that locally sourced stallions were exclusively selected for sacrifice in cemeteries. Genetic sexing of equids is increasingly common in archeological investigations (*33*–*35*), as morphological sex identification methods are often inadequate (*28*, *36*–*38*). Modern equids are not sexually dimorphic (*39*), and skeletal dimorphism is limited even further in geldings, although the extent remains unclear (*36*, *39*).

Research has also demonstrated that sacrificed horses are morphologically and metrically consistent with local breeds such as the forest tarpan [*Equus ferus* or *Equus sylvestris*; see discussion in (*40*)] and Lithuanian ž*emaitukas* breeds from the sixth century CE onward (*6*, *7*, *9*, *10*, *26*, *29*). Osteometrical analysis provides little indication that the horses are not local to the region (*7*), although horses at the higher end of stature distributions have been speculated to be examples of nonlocal breeds at sites such as Gross Ottenhagen (*25*). There is sparse historical evidence for horse trading or raiding (*41*) and limited evidence for horses sourced from the eastern Baltic region at Scandinavian sites (*42*). Imported material culture is a feature of the eastern Baltic region particularly in Viking (~850 to 1050 CE) and Late Iron Age (~1050 to 1250 CE) contexts (*11*). Given its location, it had a key position within long-distance trade networks connecting the Baltic Sea to Central Europe, the Byzantine world, and Islamic trading partners (*43*–*45*). Recent ^87^Sr/^86^Sr isotope analysis of sacrificed horses from Lithuanian cemeteries demonstrated that large horses from the late Roman and Migration period (third to seventh century CE) were nonlocal to their burial place, with ^87^Sr/^86^Sr values consistent with eastern Lithuania and southern Sweden (*46**,* *47*). To date, there is no direct evidence that horses buried in eastern Baltic cemeteries were sourced from outside of Balt territory.

^87^Sr/^86^Sr isotope analysis is an optimal method for identifying evidence for trade through exploring the origin of the horses. It is an established method in archeological mobility and migration studies to understand the movement of people and animals in the past (*47*–*49*). ^87^Sr/^86^Sr values in soil, water, and plants vary across a landscape based on the age and composition of the underlying geology (*49*), sea spray effects (*50*), anthropogenic impacts such as agricultural fertilizers (*51**,* *52*), and understanding biosphere variation is complex (*53*). ^87^Sr/^86^Sr values are therefore not a direct reflection of geologic strontium and the local range of bioavailable strontium needs to be identified as a prerequisite for archeological mobility research (*54**,* *55*). Once a local baseline range is established, then ^87^Sr/^86^Sr values from biological tissue can be compared, nonlocal individuals identified, and origins explored.

**Fig. 1.**
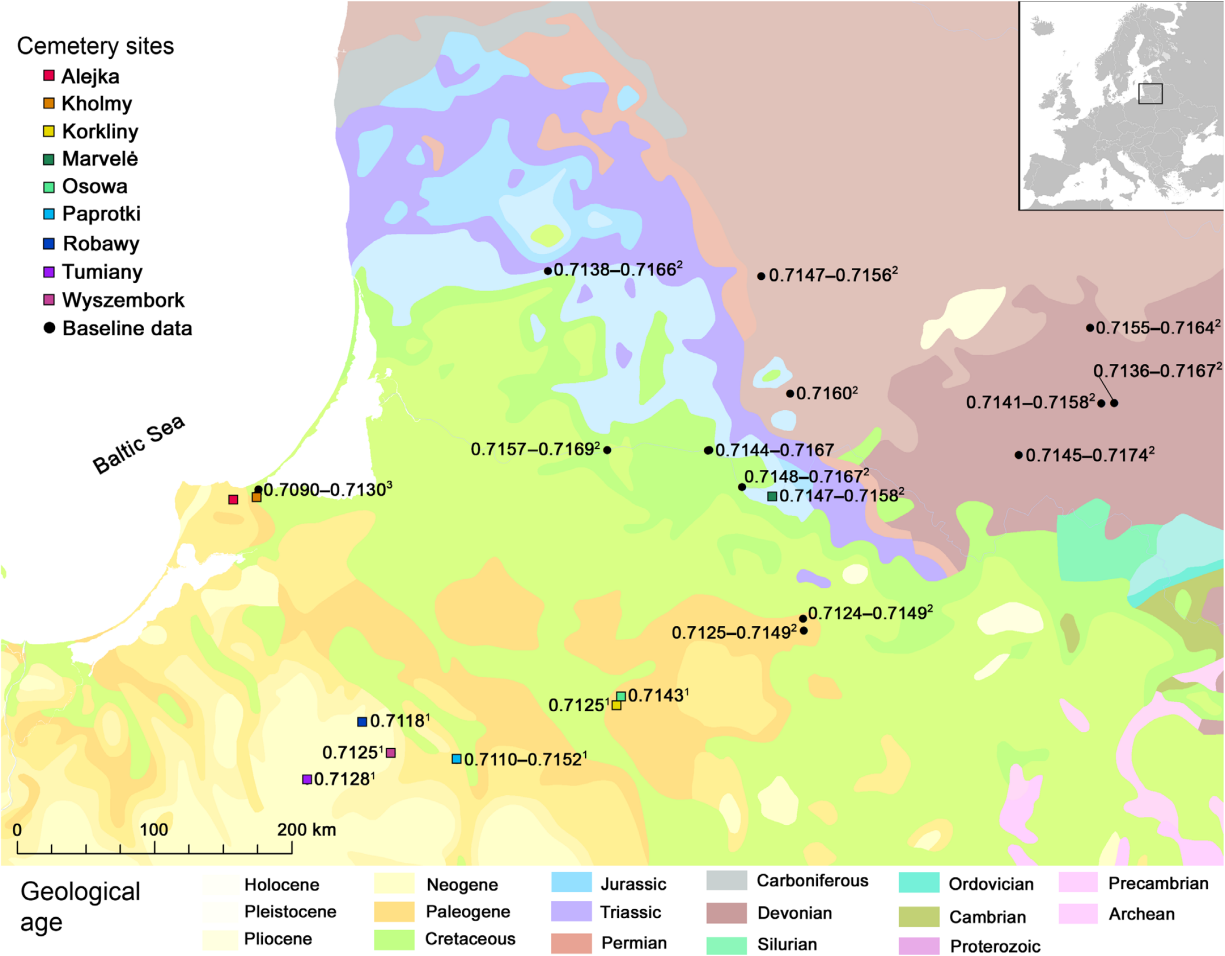
Map of the eastern Baltic region. Location of research sites and bioavailable strontium values for the eastern Baltic region. Baseline values from the following: ^1^This paper, ^2^(*46**,* *64*), ^3^figure 45 in (*59*); (*60*). Basemap, with simplified gradations in the legend (*56*).

Here, we report a biomolecular investigation of sacrificial horses from nine sites in the eastern Baltic region spanning the 1st to 13th centuries CE [modern Poland, Lithuania, and the Kaliningrad region of Russia; Fig. 1]. The study included genetic sex determination, ^87^Sr/^86^Sr isotope analysis, and radiocarbon dating of skeletal tissue from whole horse deposits buried within cemeteries, but separately from human graves. Our objective is to reevaluate the accepted belief that all sacrificed horses were male and locally sourced and if patterns change over time. Tracking horse mobility across this complex period addresses the fundamental theme of interaction between Christian and pagan communities and how, if at all, ritual behavior was mediated through these interactions.

## RESULTS

### Genetic sex analysis

All samples submitted for genetic analysis had adequate DNA preservation for polymerase chain reaction (PCR) amplification of the sex chromosome fragments and returned reliable results. Twenty-six of 76 (34%) samples submitted for genetic analysis were identified as mares (Table 1).

**Table 1.** Summary of results Genetic and ^87^Sr/^86^Sr isotope analysis results from horse teeth samples organized by cemetery site.

	DNA			^87^Sr/^86^Sr					
Site	*n* =	Male	Female	*n* =	Min	Max	Median	Mean	SD
Alejka	1	1	-	5	0.71123	0.72313	0.71397	0.71524	0.00458
Kholmy	1	-	1	6	0.71288	0.72279	0.71383	0.71517	0.00379
Korkliny	1	1	-	2	0.71385	0.71391	-	0.71388	0.00004
Marvelė	20	15	5	35	0.71445	0.73823	0.71603	0.71667	0.00390
Osowa	1	-	1	1	0.71389	-	-	-	-
Paprotki	11	7	4	8	0.71005	0.71410	0.71323	0.71258	0.00154
Robawy	8	5	3	16	0.71192	0.71493	0.71338	0.71332	0.00093
Tumiany	33	20	13	37	0.70882	0.71638	0.71397	0.71361	0.00166
Wyszembork	2	2	-	5	0.71270	0.71459	0.71342	0.71360	0.00080

### Bioavailable strontium isotope baseline for the eastern Baltic region

Modern tree leaf samples were collected from the vicinity of all sites in northeastern Poland to establish the regional ^87^Sr/^86^Sr baseline (Fig. 1). All sites are located on Cenozoic era soils, typically Quaternary glaciogenic till soils (*56**,* *57*) that returned values between 0.71102 and 0.71644 (table S2). Baseline ^87^Sr/^86^Sr values for the remainder of the Balt territory were taken from published literature (table S3). It was not possible to sample near the Kaliningrad cemeteries due to geopolitical conditions. We used the ^87^Sr/^86^Sr range from the site of Wiskiauten, located 2 km north of Kholmy (*58*). Bioavailable strontium was determined by sampling soil (*n* = 4), humans (*n* = 4), and archeological fauna (*n* = 49) [figure 45 in (*59*); (*60*)]. Published ^87^Sr/^86^Sr values for Marvelė and three surrounding sites were also used to develop a local baseline for the Kaunas region (*46*). Assembled ^87^Sr/^86^Sr baseline values demonstrate the broad territory of the Balts in modern Poland, Kaliningrad, and Lithuania has a very wide range from 0.709 to 0.717, thus reducing the potential of this method to identify horses nonlocal to the region.

### Horse mobility

**Fig. 2.**
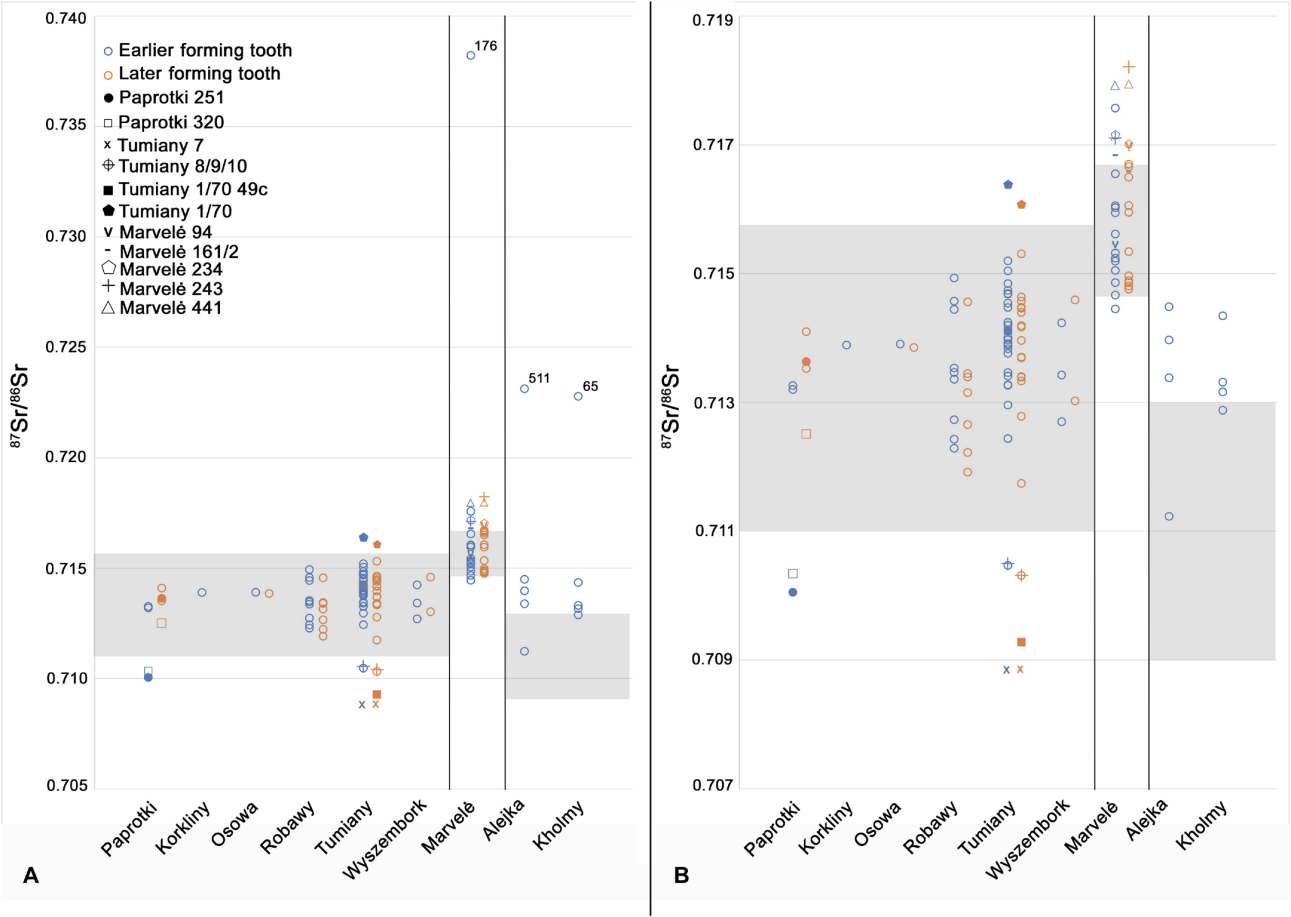
Scatterplot of ^87^Sr/^86^Sr results. ^87^Sr/^86^Sr results from early and later forming horse teeth from all project sites. Gray-shaded areas depict the local strontium baseline. The baseline for Marvelė includes a range for the Kaunas region from Piličiauskienė *et al*. (*46**,* *64*). Baseline for Alejka and Kholmy from the nearby site of Wiskiauten [figure 45 in (*59*); (*60*)]. Individuals with one or more ^87^Sr/^86^Sr values falling outside of the local baseline are identified with a special character (upper left). (**A**) ^87^Sr/^86^Sr results including highly radiogenic outliers. (**B**) ^87^Sr/^86^Sr results without highly radiogenic outliers.

^87^Sr/^86^Sr values from horse teeth samples identified several individuals reared outside of their burial area, but most (*n* = 71 individuals) have values consistent with the broad territory of the Balts (between 0.709 and 0.717). One individual from Central Lithuania (Marvelė 176) and two individuals from Kaliningrad (Alejka 511 and Kholmy 65) returned highly radiogenic, outlier values (Fig. 2A) and are discussed in the next section.

When possible, enamel from an early forming and late forming tooth (typically first and third molars) was sampled from the same individual to track mobility between birth and the start of their working lives (*61*). Two horses dated to the Roman period, Paprotki 251 and 320, were not born locally but had moved to the region before reaching skeletal maturity (Fig. 2B). The ^87^Sr/^86^Sr values from the first molars (0.71032 and 0.71005) are documented nearby on the Sambia Peninsula (*59*) and southern Poland (*62*) and may represent trading or raiding with neighboring groups or the seasonal home range of a feral horse of this stature (*63*). Migration period horses from Tumiany show even greater mobility, with five horses identified as nonlocal (Fig. 2B). Tumiany 7 had the least radiogenic ^87^Sr/^86^Sr value in the study (0.7088 ± 9.26 × 10^−6^), but again consistent with neighboring regions in southern Poland (*62*). Tumiany 1/70 had nonlocal ^87^Sr/^86^Sr values consistent with a juvenile period spent in Central Lithuania (Fig. 1 and table S3).

Four nonlocal horses from Viking or Iron Age contexts in Marvelė had at least one ^87^Sr/^86^Sr value between 0.717 and 0.719. This is similar to data reported from the Migration period sites of Paduobė-Šaltaliūnė and Pagrybis and consistent with both eastern Lithuania and southern Sweden (*46**,* *64*).

Eight of 10 individuals sampled from Alejka and Kholmy were more radiogenic than the baseline from Wiskiauten, with even the median ^87^Sr/^86^Sr values falling above the baseline (Table 1). One explanation is that the Wiskiauten baseline is not representative of the local range at Alejka and Kholmy. Given the baseline values from neighboring Poland, it is possible that a more extensive sampling program would return local ^87^Sr/^86^Sr values as high as 0.715, encompassing all nonlocal individuals except for the highly radiogenic Alejka 511, discussed in the next section. This is considered unlikely, given the sites are in relatively equivalent Paleogene-Cretaceous lithological zones and similar distances to the coast. Wiskiauten is more than 2500 m from the modern Baltic Sea coast (*65*), and therefore, the impact of sea spray reducing biogenic Sr values can be discounted (*50*). Additional sampling is required to clarify the local baseline around Alejka and Kholmy to determine whether ^87^Sr/^86^Sr values between 0.713 and 0.715 should be considered consistent with “local” values.

### Origin and dating of long-distance imported horses

Three horses returned highly radiogenic ^87^Sr/^86^Sr values inconsistent with the eastern Baltic region: Marvelė 176 (0.73823), Alejka 511 (0.72313), and Kholmy 65 (0.72279). Using the method by Hoogewerf *et al*. (*66*), we determined the statistical likelihood that the horses spent their juvenile period in different areas of Europe. Results confirm that there is no possibility that the horses originated in the territory of the Baltic tribes and that the region of the highest likelihood for these horses is the Fennoscandian Peninsula, specifically east-central Sweden or southern Finland (Fig. 2A).

Calibrated radiocarbon dating results demonstrate Marvelė 176 was sacrificed 1115 ± 80 CE and Alejka 511 around 1225 ± 50 CE (table S4). Kholmy 65 was not directly dated; based on the poor grave goods dating from adjacent burials, it can be dated either from the 8th/9th to 10th centuries or from the mid-11th to mid-12th centuries (table S4). Therefore, all examples of long-distance imported horses deposited in Central Lithuania and the north coast of the Sambia Peninsula are broadly contemporary and date to the Viking or Late Iron Age.

## DISCUSSION

### Variable sex selection

Genetic results prove that mares were sacrificed and deposited in Baltic cemeteries consistently from the Roman Period through the Late Iron Age. Genetic testing cannot determine whether male horses were stallions or geldings, but we can state they are more common than mares, representing 66% of all sacrifices in our sample. This imbalance could be due to the relative economic importance of mares to the elite Balts who bred horses and consumed fermented horse milk (*67*). The site of Poganowo IV (*30*) remains an outlier due to its exclusive evidence of horse sacrifice outside of human cemetery sites, but the site can no longer be considered an outlier due to the equal sex ratio of the sacrifices, which is not substantively different to the evidence found in sampled cemeteries.

A recent study of horse sacrifices in Iceland found that male horses were overwhelmingly chosen for sacrificial offerings, interpreted as evidence that the purpose of the ritual performance was to represent characteristics assigned to the stallion, namely, aggressiveness or virility (*35*). This cannot be argued for Balt horse sacrifices in any period or tribal territory. Marvelė 176 is the only Fennoscandian import with sufficiently preserved tissue to submit for genetic analysis and was determined to be male. However, a single sample is insufficient to determine whether males were preferentially imported.

### ^87^Sr/^86^Sr baseline for northeastern Poland

Bioavailable strontium values from modern tree leaf sampling in northeastern Poland range from 0.71102 to 0.71518 (table S2). Minimum and maximum values were both sampled from within a kilometer at Paprotki suggesting that local variability is high throughout this landscape. For this reason, we considered the local range for northeastern Poland (Warmian-Masurian Voivodeship) conservatively at 0.7110 to 0.7155. This is a wide baseline for relatively recent geological deposits. However, the project area encompasses Quaternary glaciogenic till soils with heterogeneous parent materials (*57*). It is expected that mixed-age parent material returns broad ^87^Sr/^86^Sr values. Frei and Frei (*68*) similarly observed that glacially deposited Precambrian granitoids in Denmark will produce radiogenic “hotspots” created by local outliers emphasizing the potential diversity of glaciogenic soils and the need for a comprehensive sampling strategy.

### Origins of sacrificed horses

Horses sacrificed during the Roman (~1 to 450 CE) and Migration (~450 to 700 CE) periods were typically locally sourced, and there was a marked increase in nonlocally sourced horses during the Viking Age and Late Iron Age (Fig. 2A). This includes three individuals definitively sourced from outside of Balt territory, procured from Christian neighbors.

**Fig. 3.**
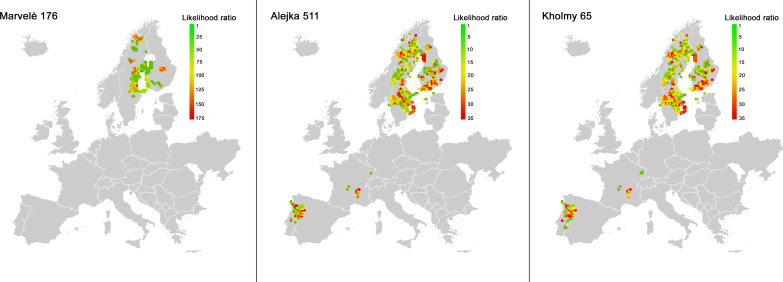
Map of potential outlier horse origins. Likelihood ratio (LR) for geographic origins of highly radiogenic horse samples using method and model from Hoogewerff *et al*. (*66*). LR is the probability of the analytical match using calculated uncertainty divided by the probability of observing the grid cell in the GEMAS dataset.

Although eastern Sweden and Central Finland are the most likely candidates for the origin of the horses, we considered whether horses may have been brought to the region by the Germanic crusading orders. Historical sources document that the indigenous Prussians acquired horses while fighting with the Teutonic Order, who introduced larger-stature warhorses to the eastern Baltic region (*69*, *70*). There is no possibility that Marvelė 176 originated anywhere in modern Germany, but there is a possibility that Alejka 511 and Kholmy 65 could have originated in southern Germany where there are Precambrian granites with ^87^Sr/^86^Sr values approximating 0.72 (*71*), but sampling has proven that the soils in the adjacent valleys have much lower ^86^Sr/^87^Sr values, typically ≤0.710. It is unlikely that either feral or managed horses would consume resources entirely from the mountainous region without notable input from grazing on valley grass. This corresponds to the low probability of an Alsatian origin for Alejka 511 and Kholmy 65 and zero probability for Marvelė 176 to originate from this region using the Geochemical Mapping of Agricultural and Grazing land Soil (GEMAS) dataset (Fig. 3) (*66*). Therefore, it is highly likely that all horses originated from the Fennoscandian Peninsula and their introduction to the eastern Baltic region was unrelated to the Crusades.

There are additional considerations with using the method by Hoogewerff *et al*. (*66*) in this research context. Results from a single isotope proxy are easy to overinterpret and should typically be used to exclude, rather than suggest geographic origins (*49**,* *72*). However, the values from Marvelė 176, Alejka 511, and Kholmy 65 are so atypical that most of Western Europe is easily excluded. Furthermore, there is strong archeological and historical evidence for close connections with eastern Sweden in these periods (*42*, *44*) that correspond well with the results of the likelihood ratios. Concerns remain, however, with the GEMAS data used to create the model by Hoogewerff *et al*. (*66*). GEMAS data are samples from grazing and agricultural soils, which are less reliable than archeological fauna teeth or modern plant sampling for establishing a bioavailable strontium baseline (*53**,* *54*). In addition, this dataset does not include non-European Union neighboring regions where the horses may have originated, including the Kaliningrad region, western mainland Russia, or Belarus. Even across Western Europe, the sampling grid resulted in generally low-resolution data compared to local site sampling which leads to missing variation in the landscape. This is key for our area of interest, as the highest standard error estimates for combined measured and modeled ^87^Sr/^86^Sr values occur in central Sweden and Central Finland due to the predicted diversity of expected ^87^Sr/^86^Sr values in older geologies (*73*).

The three individuals with strontium isotope ratios above 0.72 are unequivocally nonlocal horses, but any of our samples may also be of Fennoscandian origin given the level of variation in bedrock geology on the Fennoscandian Peninsula, with reported local values ranging from 0.708 in Scania (*74*) to 0.745 in Finland (*75*). Our results cannot prove that individual horses were raised locally but can only exclude locations of geographic origin (*49*). When local bioavailable strontium ranges overlap, the geographic origin cannot be determined. The addition of further isotope proxies has the potential to identify other nonlocal animals (*72*).

### Long-distance horse trade

A key question is how Fennoscandian horses were acquired. Cross-Baltic trade networks were well-established by the 11th century CE (*44*), but Viking-style piracy and raiding are also documented (*76*). In archeological context, Marvelė 176 does not differ from the neighboring horse graves either in terms of artefactual context or typology, but it is worth noting that the Marvelė cemetery is located in the network of regional connections of the East Baltic, and there are clear signs of Viking activity (*77*). Alejka 511 was buried with a weight, a key item to traders at the time seen only on Scandinavian-influenced trade sites from the period (*11*, *42*). This suggests that whoever owned the horse was pagan-identifying and an active participant in cross-Baltic trade. Sampled horses were not buried associated with specific graves, so it is beyond the interpretive potential of this study to determine whether imported horses traveled with an owner or were acquired by an indigenous Balt. There is isotopic evidence that individuals traveled across the North Sea and were buried with Scandinavian-raised horses in the Early Viking Age (*78*).

Radiocarbon dates confirm that these horses post-date the official Swedish kingdom’s conversion to Christianity (~1000 CE) but coincide with an internal pagan resistance that persisted throughout the 11th century (*79*). It is less simple to date Finnish conversion, also seen to be a gradual process occurring between the 10th and 13th centuries CE (*80*), although church infrastructure was slower to develop (*81*), and there is evidence for furnished graves into the 14th century in the Karelia region (*82*). Sámi populations in Arctic Finland likely practiced a syncretic religion into the 17th century (*83*). We can therefore not discount the possibility that the Fennoscandian horses identified at Marvelė, Alejka, or Kholmy represent horses who traveled with and were sacrificed upon the death of their Scandinavian or Finnish owner, who was themselves buried in the pagan style of the Balts. Alternately, these horses provide direct evidence for purported cross-Baltic horse trading or raiding starting during the Viking Age (*41*). In either case, our results prove that horses were crossing the Baltic Sea on ships, a level of mobility not previously recognized archeologically. Additional research characterizing human and horse mobility in the eastern Baltic region during the Viking Age is warranted, aided by the strontium isoscape mapping presented here. Additional sampling is required on the Sambian Peninsula to further understand bioavailability in the regional isoscape.

Biomolecular methods have proven to be key for testing the efficacy of traditional osteological methods and examining the underlying assumptions of well-traveled archeological narratives. Our results established that mares were commonly chosen for public sacrifice in all periods and that the presence of canine teeth is inadequate to distinguish sex in equids. We further demonstrate that the Balts sourced and sacrificed horses from neighboring lands undergoing gradual conversion themselves. While the socioeconomic prestige of imported animals may have been the essential characteristic of this sacrificial choice, this may also be viewed as a powerful act of resistance and resilience within a pagan-persisting community.

## MATERIALS AND METHODS

### Sample

The project generated biomolecular data from 80 individual horses from nine sites (Fig. 1 and table S1) and produced a bioavailable strontium dataset from seven sites in northeastern Poland (Fig. 1 and table S2). A total of 115 horse teeth were extracted for ^87^Sr/^86^Sr analysis from 74 individual horses (table S4). When possible, the first and third permanent molars were sampled, as these represent the earliest (0.5 to 23 months) and last mineralization events (21 to 55 months) (*61*). For DNA and accelerator mass spectrometry (AMS) radiocarbon dating, dentine was cleaned and prepared for DNA and collagen extraction from the same tooth (typically M1) sampled for ^87^Sr/^86^Sr. The genetic analysis included 76 samples from 70 individual horses. AMS dating included three samples from individual horses from Marvelė and eight samples from horses and associated material from Alejka and Kholmy.

To establish the baseline bioavailable strontium for cemeteries, leaves were sampled from moderate-rooted trees located at least 50 m from agricultural fields and water and within 1 km of cemetery sites (table S2). A mixed sampling approach was followed for research sites Tumiany, Osowa, Korkliny, Robawy, and Wyszembork. We sampled the leaves of three species from different locations around the site. Approximately 100 mg from each plant species was mixed to create a single sample for ^87^Sr/^86^Sr analysis. A single plant sampling approach was conducted at Paprotki to quantify the range of variation in the glaciogenic landscape. Six species were sampled from around Paprotki cemetery and settlement, but the samples were analyzed separately to give six distinct values.

### Strontium isotope analysis

Sample preparation for strontium analysis was undertaken at Cardiff University BioArchaeology laboratory. Enamel samples (20 to 50 mg) were cut from the mesiobuccal tooth cusp within 1 cm of the occlusal surface and cleaned using a diamond saw and burr to remove the outer cementum, at least 10 μm of the enamel surface, and all adhering dentine. Enamel samples were cleaned in an ultrasonic bath in deionized water and dried. Plant samples were freeze-dried, coarsely crushed, and weighed to approximately 300 mg per sample (100 mg per plant species for sites with a mixed sample approach).

Sample chemistry was conducted at the Cardiff Earth Laboratory for Trace Element and Isotope Chemistry. Enamel samples were digested overnight in 8 M HNO_3_ on a hotplate at 120°C. Plant leaves were placed in distilled HNO_3_ and H_2_O_2_ and digested using the Milestone Ethos Easy microwave digestion system. The sample was spun down using a microcentrifuge to isolate residue and the supernatant was transferred into Savillex beakers. Following preparation, enamel and plant samples followed the strontium extraction protocol in (*84*). Approximately 100 μl of pre-cleaned Eichrom Sr-Spec resin is loaded into extraction columns. Digested samples were then loaded into the resin columns. Matrix elements, including Ca and trace Rb, were removed in several washes of 8 M HNO_3_ before Sr was eluted and collected in 0.05 M HNO_3_. Samples are dried overnight on a hotplate at 120°C, and then the process is repeated for a second time for the effective removal of any remaining traces of Ca. Once dry, purified samples were redissolved in 0.3 M HNO_3_.

The ^87^Sr/^86^Sr ratios were measured using a Nu Plasma II multi-collector inductively coupled plasma mass spectrometer at Cardiff University. Samples were introduced using an Aridus II desolvator introduction system. All data were first corrected for on-peak blank intensities, then mass bias was corrected using the exponential law and a normalization ratio of 8.375209 for ^88^Sr/^86^Sr (*85*). Residual krypton (Kr) and rubidium (^87^Rb) interferences were monitored and corrected for using ^82^Kr and ^83^Kr (^83^Kr/^84^Kr = 0.20175 and ^83^Kr/^86^Kr = 0.66474; without normalization) and ^85^Rb (^85^Rb/^87^Rb = 2.5926), respectively. Analysis of NIST SRM 987 during the analytical session gave an ^87^Sr/^86^Sr value of 0.710292 ± 0.000007 (2σN, *n* = 11), and all data are corrected to NIST SRM 987 values of 0.710248 (*86*). Total procedural blanks are typically less than 20 pg of Sr, which is negligible relative to the Sr in samples (greater than 20 ng). Accuracy of the NIST SRM 987 normalization and the chemistry processing was assessed by repeat measurements of ^87^Sr/^86^Sr ratio in NIST SRM 1400 (bone ash, processed through chemistry similar to the unknown samples), giving an average ^87^Sr/^86^Sr ratio of 0.713111 ± 0.000014 (2σN, *n* = 5), which is consistent with the published value (0.713126 ± 0.000017) (*87*). The long-term reproducibility of the ^87^Sr/^86^Sr ratio measurements is testified with repeat measurements of the in-house standard NEB-5-Sr, a bulk dissolution of a well-preserved ~300,000-year-old fossilized *Favidae* coral from Henderson Island, South Pacific (*88*). The NEB-5-Sr standard is processed through column chemistry and measured, interspersed with unknown samples. The NEB-5-Sr ^87^Sr/^86^Sr ratio measurements yielded 0.709162 ± 0.000017 (2 SD on 145 measurements from the period March 2021 to December 2022), an ^87^Sr/^86^Sr ratio in the range of average seawater ~300 thousand years ago.

### aDNA

Teeth were prepared for aDNA extraction by briefly sterilizing under ultraviolet light, removing the outer layer of tissue, and powdering the exposed, clean tissue. Isolation of aDNA from the prepared powdered tissues (approximately 0.1 g of powder per sample) was performed with a kit for the trace amounts of DNA isolation - Sherlock AX Direct (mod.1; A&A Biotechnology, Gdańsk, Poland) in accordance with the protocol provided by the manufacturer. The obtained nucleic acid was eluted in 50 μl of 10 mM tris (pH 8.5) and quality-verified by checking its concentration and purity on the NanoDrop 2000 spectrophotometer (Thermo Fisher Scientific, USA). The isolated aDNA samples were stored at −20°C.

Three fluorescently labeled probes were used for the amplification of X and Y chromosome fragments. The probes were designed using GenBank and Primer Express (Thermo Fisher Scientific). The first one (NED) complementary to the X chromosome part (*PLP1* gene, ENSECAG00000015446) was used in the amplification with primers F: GTCAGGCCAAGGAGAGTAGCA; R: GCACATCCTCCTCCACTTATGC; and probe sequence NED: CCCAGTTCTTAGGTCACA using the TaqMan Gene Expression Master Mix kit (Thermo Fisher Scientific). The other two probes (VIC and FAM) were complementary to the Y chromosome fragment and included simultaneously polymorphism described previously as distinguishing *Equus caballus* from *Equus przewalskii* (MH341179.1, g731821T > C) (*89*). Amplification of the Y chromosome part was performed with primers F: AGACCCGCCGGTGC; R: CAATTCCCTGGAGCCTCTGTAG and probes sequences VIC: AGTCCTGGTGAATGAG; FAM: TCCTGGCGAATGAG using TaqPath ProAmp Master Mix (Thermo Fisher Scientific). Real-time PCR reactions were performed on the QuantStudio 7 Flex instrument (Thermo Fisher Scientific). Samples presenting X and one of Y signals were recognized as male, while samples only with the X signal were recognized as female.

### C14 dating

Direct AMS radiocarbon dates from Marvelė horse teeth were obtained from the commercial laboratory ^14^Chrono Centre, Queens University Belfast, following standard protocols for collagen extraction (14chrono.org). Eight horse teeth, human cremated bones, and charcoal samples from Kholmy and Alejka-3 were submitted for dating at the Leibniz Laboratory for Radiometric Dating and Stable Isotope Research of CAU, Kiel, following standard protocols for apatites and collagen extraction (bones) and acid-base-acid pretreatment (charcoal) (*90*). Extracts were combusted, graphitized, and measured by AMS, following established procedures (*91*). All results were calibrated using the IntCal20 curve (*92*). Radiocarbon age and calibrated age (±2 SD) are reported in table S4.

## Supplementary Materials

### This PDF file includes:

Tables S1 to S3 Legend for table S4 References
