# Supplementary material for: Biomolecular evidence reveals mares and long-distance imported horses sacrificed by the last pagans in temperate Europe
Source: Sci Adv. 2024 May 17;10(20):eado3529. doi: 10.1126/sciadv.ado3529 (PMC11100556; doi:10.1126/sciadv.ado3529)
Supplement: Supplementary file 1 — Tables S1 to S3 Legend for table S4 References [file sciadv.ado3529_sm.pdf]

Science Advances
AAAS

Supplementary Materials for

Biomolecular evidence reveals mares and long-distance imported horses sacrificed by the last pagans in temperate Europe

Katherine M. French *et al.*

Corresponding author: Richard Madgwick, madgwickRD3@cardiff.ac.uk

Sci. Adv. **10**, eado3529 (2024)
DOI: 10.1126/sciadv.ado3529

This PDF file includes:

Tables S1 to S3
Legend for table S4
References

Other Supplementary Material for this manuscript includes the following:

Table S4

Table S1.
Location of all sites included in study and description of samples taken.

Country	Site	Period	Archaeological Culture	Number of Individual Horses	aDNA Samples (n=)	Sr Samples (n=)	Source of Baseline Strontium Data
Poland	Korkliny	Migration	Sudovian	1	1	2	This project - mix of 3 modern plant samples
Poland	Osowa		Sudovian	1	1	1	This project - mix of 3 modern plant samples
Poland	Paprotki Kolonia	Roman	Bogaczewo	10	10	8	This project - 6 modern plant samples; 3 from near settlement, 3 from near cemetery
Poland	Robawy	Migration	Olstzyn	9	8	16	This project - mix of 3 modern plant samples
Poland	Tumiany	Migration	Olstzyn	26	33	37	This project - mix of 3 modern plant samples
Poland	Wyszembork IV	Roman<br>Migration	Bogaczewo<br>Olstzyn	2	2	5	This project - mix of 3 modern plant samples
Lithuania	Marvelė	Roman<br>Migration<br>Viking	Aukštaičiai	20	19	35	(46-47)
Russia	Alejka-3	Roman LIA	Dollkeim-Kovrovo Prussian	5	1	5	Baseline data for nearby site of Wiskiauten (60, 61: Fig. 45)
Russia	Kholmy	Roman LIA	Dollkeim-Kovrovo Prussian	6	1	6	Baseline data for nearby site of Wiskiauten (60, 61: Fig. 45)
			TOTAL	80	76	115	

Table S2.
Modern plant samples for site baseline.

Country	Site	Species	Common Name	$^{87}Sr/^{86}Sr$	SE (2 SD)
Poland	Tumiany	*Alnus sp.*<br>*Betula sp.*<br>*Salix sp.*	Alder<br>Birch<br>Willow	0.71277	6.84E-06
Poland	Osowa	*Corylus avellana*<br>*Carpinus betulus*<br>*Quercus robur*	Hazel<br>Hornbeam<br>Oak	0.71434	7.68E-06
Poland	Korkliny II	*Syringa vulgaris*<br>*Quercus robur*<br>*Sorbus sp.*	Lilac<br>Oak<br>Rowan	0.71249	7.72E-06
Poland	Robawy	*Betula sp.*<br>*Solidago virgaurea*<br>*Salix sp.*	Birch<br>Goldenrod<br>Willow	0.71175	7.20E-06
Poland	Wyszembork IVa	*Solidago virgaurea*<br>*Salix sp.*<br>*Tilia sp.*	Goldenrod<br>Willow<br>Lime Tree	0.71250	7.52E-06
Poland	Paprotki Settlement	*Tilia sp.*	Lime	0.71428	7.66E-06
Poland	Paprotki Settlement	*Quercus robur*	Oak	0.71375	1.05E-05
Poland	Paprotki Settlement	*Juglans sp.*	Walnut	0.71518	5.50E-06
Poland	Paprotki Cemetery	*Sambucus nigra*	Black Elderberry	0.71102	7.78E-06
Poland	Paprotki Cemetery	*Prunus domestica*	Plum	0.71151	8.56E-06
Poland	Paprotki Cemetery	*Syringa vulgaris*	Lilac	0.71152	1.07E-05

Table S3.

Comparative baseline data for Eastern Baltic and neighboring regions.

Country	Site	Lat	Long	Sample Type	Baseline Min	Baseline Max	Reference
Estonia	Northern coast	59.5025	25.516944	Small Mammals	0.7106	0.7159	(93)
Estonia	Maaremaa, Salme	58.183611	22.245833	Modern, Archaeological Fauna	0.7104	0.7139	(60)
Finland	Bothnian Bay	65.334167	25.366667	Modern Plant	0.7178	0.7347	(94)
Finland	North Finland			Modern Plant, Water	0.717	0.7385	(75)
Finland	South Finland			Modern Plant, Water	0.7295	0.745	(75)
Germany	Pre-Alpine Lowlands	48.084167	9.681944	Archaeological Fauna Teeth (pig)	0.7072	0.7103	(71)
Germany	Southern Rhine Valley	49.825	8.496111	Archaeological Fauna Teeth (pig)	0.7083	0.7107	(71)
Germany	Bavarian Forest	49.524167	12.416389	Archaeological Fauna Teeth (pig)	0.7189	0.7221	(71)
Latvia	Riga	56.948889	24.104722	Archaeological Fauna	0.712	0.7125	(95)
Lithuania	Alytus	54.395278	24.045556	Archaeological Fauna Teeth	0.7125	0.7149	(46)
Lithuania	Antilgė	55.5325	25.92	Archaeological Fauna Teeth	0.7155	0.7164	(46)
Lithuania	Bakšiai	54.439167	24.0425	Archaeological Fauna Teeth	0.7124	0.7149	(46)
Lithuania	Daučionys	55.076111	23.428333	Archaeological Fauna Teeth	0.7144	0.7167	(46)
Lithuania	Dubingiai	55.057778	25.452778	Archaeological Fauna Teeth	0.7145	0.7174	(46)
Lithuania	Garniai	55.075833	23.418611	Archaeological Fauna Teeth	0.7167	0.7185	(46)
Lithuania	Jurbarkas	55.076667	22.760278	Archaeological Fauna Teeth	0.7157	0.7169	(46)
Lithuania	Kaltanėnai	55.251389	25.993056	Archaeological Fauna Teeth	0.7141	0.7158	(46)
Lithuania	Kaunas	54.897778	23.904167	Archaeological Fauna Teeth	0.7147	0.7158	(46)
Lithuania	Kėdainiai	55.28722	23.9575	Archaeological Fauna Teeth	0.7160	-	(46)
Lithuania	Kretuonas	55.252222	26.078333	Archaeological Fauna Teeth	0.7136	0.7167	(46)
Lithuania	Kulautuva	54.937778	23.6425	Archaeological Fauna Teeth	0.7148	0.7167	(46)
Lithuania	Varniai	55.5325	25.920278	Archaeological Fauna Teeth	0.7138	0.7166	(46)

Lithuania	Žemaitiškė	55.252222	26.078333	Archaeological Fauna Teeth	0.7154	0.7155	(46)
Lithuania	Žilionys	55.723333	23.768889	Archaeological Fauna Teeth	0.7147	0.7156	(46)
Poland	Wyszembork Iva	53.9232	21.34944	Modern Plant	0.712495	-	This paper
Poland	Tumiany	53.81807	20.7967	Modern Plant	0.712768	-	This paper
Poland	Robawy	54.04211	21.15721	Modern Plant	0.711753	-	This paper
Poland	Paprotki Cemetery	53.89951	21.77515	Modern Plant	0.711021	0.711517	This paper
Poland	Paprotki Settlement	53.8986	21.78584	Modern Plant	0.713748	0.715182	This paper
Poland	Osowa Settlement	54.16758	22.85297	Modern Plant	0.714341	-	This paper
Poland	Korkliny II	54.11211	22.82484	Modern Plant	0.712488	-	This paper
Poland	Załęcze Landscape Park	51.093781	18.685317	Modern Plant	0.712534	0.712614	(96-97)
Poland	Drawa National Park	53.071778	15.93625	Modern Plant	0.712712	0.712774	(96-97)
Poland	Krzesin Landscape Park	52.045139	14.855128	Modern Plant	0.711438	0.711611	(97)
Poland	Bolkowo	53.900528	16.065583	Modern Plant	0.711312	0.7136	(97)
Poland	Barycz Valley Landscape Park	51.574564	17.401617	Modern Plant	0.711926	0.712716	(97)
Poland	Koszyce	50.1	20.458611	Animal Teeth (pig)	0.7104	0.7114	(63)
Poland	Drawsko	53.518333	15.9025	Modern Fauna Bone	0.7082	0.7121	(98)
Russia	Wiskiauten	54.928333	20.478056	Archaeological Fauna Teeth	0.709	0.713	(60-61)
Sweden	Scania - Area 1	56.378056	13.992222	Modern Plant, Lake Water, Soil Leachate	0.7123	0.7245	(74)
Sweden	Scania - Area 2	55.870278	13.889444	Modern Plant, Lake Water, Soil Leachate	0.7097	0.7183	(74, 99)
Sweden	Scania - Area 3/4	55.5425	13.306667	Modern Plant, Lake Water, Soil Leachate	0.708	0.714	(74)
Sweden	Gotland-wide	57.478611	18.481944	Small Mammals, Modern Plants, Soil	0.7098	0.7128	(100)
Sweden	Mälaren region			Small mammals, Fauna (sheep/goat, dog)	0.723	0.7333	(101)
Sweden	Falköping Area	58.164524	13.454738	Archaeological Fauna	0.71268	0.72463	(102)

Sweden	Varnhem/Borgunda	58.309498	13.80962	Archaeological Fauna	0.71551	0.71755	(103)
Sweden	Forshem/Skara	58.385228	13.439759	Archaeological Fauna	0.71416	0.72918	(102)
Sweden	Länghem	57.649918	13.279574	Archaeological Fauna	0.71781	0.71894	(102)
Sweden	Södra Ving	57.840319	13.308931	Archaeological Fauna	0.71535	-	(102)
Sweden	Bornholm (north half of island)	55.218333	14.932778	Archaeological Fauna	0.7114	0.7231	(104)
Sweden	Öland			Modern and Archaeological Fauna Teeth	0.7109	0.7164	(105)

Table S4.
Results of DNA, strontium, and radiocarbon dating analysis.

<Oversized table. Uploaded separately as Table S4.xlsx>

REFERENCES

1. D. von Güttner-Sporzyński, Northern crusades: Between holy war and mission, in *The Crusader World*, A. Boas, Ed. (Routledge, 2015), pp. 144–162.

2. W. Hensche, Einiges zur Kenntniss der Todtenbestattung bei den heidnischen Preussen. *Schrift. Physikal.-Ökono. Gesell. Königsberg.* **2**, 131–138 (1862).

3. E. Hollack, Die Grabformen ostpreussischer Gräberfelder. *Zeit. Ethnol.* **40**, 145–193 (1908).

4. C. Engel, W. La Baume. *Kulturen und Völker der Frühzeit in Preußenlande.* (Gräfe und Unser, 1937).

5. R. Kulikauskienė, Pogrebenija s konjami u drevnich litovcev. *Sov. Archeol.* **57**, 211–222 (1953).

6. J. Jaskanis, Human burials with horses in Prussia and Sudovia in the first millennium of our era. *Acta Balt.-Slav.* **4**, 29–65 (1966).

7. M. Bertašius, L. Daugnora, Viking age horse graves from Kaunas region (Middle Lithuania). *Int. J. Osteoarchaeol.* **11**, 387–399 (2001).

8. A. Bliujienė, D. Butkus, Burials with horses and equestrian equipment on the Lithuanian and Latvian littorals and hinterlands (from the fifth to the eighth centuries). *Archaeol. Balt.* **11**, 149–163 (2009).

9. M. Karczewska, M. Karczewski, A. Gręzak, The role of horse burials in the Bogaczewo Culture. *Archaeol. Balt.* **11**, 56–88 (2009).

10. A. V. Zinoviev, Horse burials of Samland Natangen and adjacent areas in context of antique and medieval symbolic culture, *Archaeol. Lit.* **12**, 25–35 (2011).

11. R. Shiroukhov, Prussian graves in the Sambian peninsula, with imports, weapons and horse harnesses, from the tenth to the 13th century: The question of the warrior elite. *Archaeol. Balt.* **18**, 224–255 (2012).

12. A. Bliujienė, M. Stančikaitė, G. Piličiauskienė, J. Mažeika, D. Butkus, Human-horse burials in Lithuania in the late second to seventh century AD: A multidisciplinary approach. *Eur. J. Archaeol.* **20**,

682–709 (2017).

13. G. Piličiauskienė, L. Kurila, Ž. Ežerinskis, J. Šapolaitė, A. Garbaras, A. Zagurskytė, V. Micelicaitė, Horses in Lithuania in the Late Roman-Medieval Period (3rd-14th C AD) Burial Sites: Updates on size, age and dating. *Animals* **12**, 1549 (2022).

14. A. Pluskowski, *The Archaeology of the Prussian Crusade* (Routledge, ed. 2, 2022).

15. M. Bertašius, Horse burials as public ritual: Lithuanian perspectives, in *The Ritual Killing and Burial of Animals*, A. Pluskowski, Ed. (Oxbow, 2011), pp. 61–75.

16. A. Pluskowski, H. Valk, S. Szczepański, Theocratic rule, native agency and transformation: Post-crusade sacred landscapes in the eastern Baltic. *Landscapes* **19**, 4–24 (2018).

17. A. Fijałkowska, Pochówki końskie w kulturze zachodnich Bałtów-próba wyjaśnienia genezy oraz związków z wierzeniami w okresie od II do XII wne. *Argumenta Historica.* **4**, 40–49 (2017).

18. M. Bertašius, *Marvelė: Ein Bestattungsplatz mit Pferdegrabern*, II Band (Kauno Technologijos Univ., Kaunas, 2009).

19. R. Shiroukhov, "Prūsų ir kuršių kontaktai XI–XIII amžiaus pradžioje archeologijos duomenimis," thesis, Klaipėdos Universitetas, Klaipėda (2012).

20. M. Bertašius, *Marvelė: Ein Graberfeld Mittellitauens*, I Band (Kauno Technologijos Univ., Kaunas, 2005).

21. W. Wróblewski, *Aschenplätze*-the forgotten burial rituals of the Old Prussians. *Archaeol. Lit.* **7**, 221–234 (2006).

22. R. Shiroukhov, Contacts between Prussians and Curonians in the 11th-early 13th centuries, according to the archaeological data, in *Materiały do Archeologii Warmii i Mazurų*, M. Hoffmann, M. Karczewski, S. Wadyl, Eds. (Univ. of Warsaw, 2015), vol. 1, pp. 255–273.

23. K. Skvortsov, A. Khokhlov, Findings of saddles from archaeological excavations in Prussian cemetery Aleika-3 in Samland (preliminary publication). *Archaeol. Balt.* **11**, 343–346 (2009).

24. V. Lang, Riding to the afterworld: Burying with horses and riding equipment in Estonia and the Baltic rim, in *Identity Formation and Diversity in the Early Medieval Baltic and Beyond*, J. Callmer, I. Gustin, M. Roslund, Eds. (Brill, 2017), pp. 48–75.

25. R. Shiroukhov. K. Skvortsov, T. Ibsen. The Early Medieval graves of Groß Ottenhagen (Berezovka). On the way from Sambia to the middle reaches of the Niemen, *Archaeol. Balt.* **28**, 150–174 (2021).

26. W. Nowakowski, Horse burials in Roman period cemeteries of the Bogaczewo culture. *Archaeol. Balt.* **11**, 115–129 (2009).

27. M. Karczewska, M. Karczewski, Landscapes of cemeteries from the Roman and Migration periods in the Masurian Lakeland (Northeast Poland). *Archaeol. Balt.* **23**, 96–111 (2016).

28. B. Vollmerhaus, H. Roos, H. Gerhards, C. Knospe, Zur phylogenie, form und funktion der Dentes canini des pferdes. *Anat. Histol. Embryol. J. Vet. Med. Ser. C.* **32**, 212–217 (2003).

29. A. Gręzak. Groby koni na cmentarzyskach kultury bogaczewskiej, in *Kultura Bogaczewska w 20 Lat Później*, A. Bitner Wróblewska, Ed. (Państwowe Muzeum Archeologiczne, 2007), pp. 353–367.

30. M. Wyczółkowski, D. Makowiecki, 2009. Horse sacrifices in Prussia in the Early Middle Ages. Ritual area in Poganowo Site IV, Olsztyn Province (Poland). *Archaeol. Balt.* **11**, 295–304 (2009).

31. N. Vėlius, Ed. *Baltų Religijos ir Mitologijos Šaltiniai* (Mokslo ir enciklopedijų leidykla, 1996).

32. R. Balsys, Paganism of Prussian: Sacred castle tulissones, ligaschones. *Bull. Lviv. Uni. Hist. Ser.* **52**, 72–92 (2016).

33. A. Götherström, The value of stallions and mares during the Early Medieval time in upper class Svealand: Molecular sex identifications on horse remains from Vendel and Eketorp. *J. Nord. Archaeol. Sci.* **13**, 75–78 (2002).

34. M. Schubert, M. Mashkour, C. Gaunitz, A. Fages, A. Seguin-Orlando, S. Sheikhi, A. H. Alfarhan, S. A. Alquraishi, K. A. Al-Rasheid, R. Chuang, L. Ermini, C. Gamba, J. Weinstock, O. Vedat, L. Orlando, Zonkey: A simple, accurate and sensitive pipeline to genetically identify equine F1-hybrids in

archaeological assemblages. *J. Archaeol. Sci.* **78**, 147–157 (2017).

35. H. M. Nistelberger, A. H. Pálsdóttir, B. Star, R. Leifsson, A. T. Gondek, L. Orlando, J. H. Barrett, J. H. Hallsson, S. Boessenkool, Sexing Viking Age horses from burial and non-burial sites in Iceland using ancient DNA. *J. Archaeol. Sci.* **101**, 115–122 (2019).

36. S. Sisson, *The Anatomy of the Domestic Animals* (Saunders, ed. 2, 1914).

37. S. Sisson, J. D. Grossman, *The Anatomy of the Domestic Animals* (Saunders, ed. 4, 1953).

38. E. N. van Asperen, Implications of age variation and sexual dimorphism in modern equids for Middle Pleistocene equid taxonomy. *Int. J. Osteoarchaeol.* **23**, 1–12 (2013).

39. C. J. Johnstone, "A biometric study of equids in the Roman world," thesis, University of York, York (2004).

40. L. Lovász, A. Fages, V. Amrhein, Konik, Tarpan, European wild horse: An origin story with conservation implications. *Glob. Ecol. Conserv.* **32**, e01911 (2021).

41. H. Steuer, Principles of trade and exchange: Trade goods and merchants, in *Wulfstan's Voyage: the Baltic Sea Region in the Early Viking Age as Seen from Shipboard*, A. Englert, A. Trakadas, Eds. (Oxbow, 2009), pp. 294–308.

42. V. Žulkus, Armed and expected: Traders and their ways in Viking times. *Archaeol. Balt.* **8**, 310–320 (2007).

43. J. Genys, Trade routes and trade centres in Western Lithuania during the early Middle Ages. *Archaeol. Balt.* **2**, 141–154 (1997).

44. M. Mägi, 2018. *Austrvegr: The Role of the Eastern Baltic in Viking Age Communication Across the Baltic Sea*, vol. 84 of *The Northern World* (Brill, 2018).

45. J. Gruszczyński, M. Janowiak, J. Shepard, Eds. *Viking-Age Trade: Silver, Slaves and Gotland* (Routledge, 2021).

46. G. Piličiauskienė, L. Kurila, E. Simčenka, K. Lidėn, E. Kooijman, M. Kielman-Schmitt, G. Piličiauskas, The origin of Late Roman period–post-migration period Lithuanian horses. *Herit.* **5**, 332–352 (2022).

47. R. A. Bentley, Strontium isotopes from the earth to the archaeological skeleton: A review. *J. Archaeol. Method Theory* **13**, 135–187 (2006).

48. R. Bendrey, T. E. Hayes, M. Palmer, Patterns of iron age horse supply: An analysis of strontium isotope ratios in teeth. *Archaeom.* **51**, 140–150 (2009).

49. J. Montgomery, Passports from the past: Investigating human dispersals using strontium isotope analysis of tooth enamel. *Ann. Hum. Biol.* **37**, 325–346 (2010).

50. E. Alonzi, S. I. Pacheco-Fores, G. W. Gordon, I. Kuijt, K. J. Knudson, New understandings of the sea spray effect and its impact on bioavailable radiogenic strontium isotope ratios in coastal environments. *J. Archaeol. Sci. Rep.* **33**, 102462 (2020).

51. J. K. Böhlke, M. Horan, Strontium isotope geochemistry of groundwaters and streams affected by agriculture, Locust Grove, MD. *Appl. Geochem.* **15**, 599–609 (2000).

52. E. Thomsen, R. Andreasen, Agricultural lime disturbs natural strontium isotope variations: Implications for provenance and migration studies. *Sci. Adv.* **5**, eaav8083 (2019).

53. E. Holt, J. A. Evans, R. Madgwick, Strontium ($^{87}Sr/^{86}Sr$) mapping: A critical review of methods and approaches. *Earth Sci. Rev.* **216**, 103593 (2021).

54. T. D. Price, J. H. Burton, R. A. Bentley, The characterization of biologically available strontium isotope ratios for the study of prehistoric migration. *Archaeom.* **44**, 117–135 (2002).

55. J. Lewis, A. W. G. Pike, C. D. Coath, R. P. Evershed. Strontium concentration, radiogenic: ($^{87}Sr/^{86}Sr$) and stable ($\partial^{88}Sr$) strontium isotope systematics in a controlled feeding study. *STAR.* **3**, 45–57 (2017).

56. K. Asch, Data from "1:5 Million International Geological Map of Europe." European Commission,

https://data.europa.eu/data/datasets/9fd6624c-0aa7-46d4-9da3-955e558cd5f1?locale=en [deposited 21 November 2005].

57. L. Marks, A. Ber, W. Gogołek, K. Piotrowska, Geological Map of Poland 1:500,000 with Explanatory Text (Polish Geological Survey, 2006).

58. A. Sirkin, "Studien zur frühmittelalterlichen Siedlungslandschaft im Samland am Beispiel des Fundplatzes Wiskiauten (Mochovoe)," thesis, Universität Kiel, Kiel (2020).

59. T. D. Price, J. Peets, R. Allmäe, L. Maldre, N. Price, Human remains, context, and place of origin for the Salme, Estonia, boat burials. *J. Anthropol. Arch.* **58**, 101149 (2020).

60. A. Sirkin, "Studien zur frühmittelalterlichen Siedlundslandschaft im Samland am Beispiel des Fundplatzes Wiskiauten (Mochovoe)," thesis, Christian Albrecht University, Kiel (2020).

61. K. A. Hoppe, S. M. Stover, J. R. Pascoe, R. Amundson, Tooth enamel biomineralization in extant horses: Implications for isotopic microsampling. *Palaeogeogr. Palaeoclimatol. Palaeoecol.* 206, 355–365 (2004).

62. A. Szczepanek, Z. Belka, P. Jarosz, Ł. Pospieszny, J. Dopieralska, K. M. Frei, A. Rauba-Bukowska, K. Werens, J. Górski, M. Hozer, M. Mazurek, Understanding Final Neolithic communities in South-Eastern Poland. *PLOS ONE* **13**, e0207748 (2018).

63. R. Pikula, D. Zaborski, W. Grzesiak, M. Smugala, Locomotor activity analysis based on habitat, season and time of the day in Polish Konik horses from reserve breeding using the Global Positioning System (GPS). *Indian J. Anim. Res.* **54**, 494–498 (2019).

64. G. Piličiauskas, E. Simčenka, K. Lidén, J. Kozakaitė, Ž. Miliauskienė, G. Piličiauskienė, E. Kooijman, P. Šinkūnas, H. K. Robson, Strontium isotope analysis reveals prehistoric mobility patterns in the southeastern Baltic area. *Archaeol. Anthropol. Sci.* **14**, 74 (2022).

65. T. Ibsen, J. Frenzel, In search of the early medieval settlement of Wiskiauten/Mohovoe in the Kaliningrad region. *Liet. Archeol.* **36**, 47–58 (2010).

66. J. A. Hoogewerff, C. Reimann, H. Ueckermann, R. Frei, K. M. Frei, T. VanAswegen, C. Stirling, M. Reid, A. Clayton, A. Ladenberger, S. Albanese, Bioavailable $^{87}Sr/^{86}Sr$ in European soils: A baseline for provenancing studies. *Sci. Tot. Environ.* **672**, 1033–1044 (2019).

67. J. Bately, Wulfstan's Voyage and his description of Estland: The text and the language of the text, in *Wulfstans' Voyage*, A. Englert, A. Trakadas, Eds. (Viking Ship Museum, 2009), pp. 15–16.

68. K. M. Frei, R. Frei, The geographic distribution of strontium isotopes in Danish surface waters—A base for provenance studies in archaeology, hydrology and agriculture. *App. Geochem.* **26**, 326–40 (2011).

69. S. Ekdahl, Horses and crossbows: Two important warfare advantages of the Teutonic Order in Prussia, in *The Military Orders. Volume II: Welfare and Warfare*, H. Nicholson, Ed. (Routledge, 1998), pp. 119–151.

70. A. Pluskowski, K. Seetah, M. Maltby, R. Banerjea, S. Black, G. Kalninš, Late-medieval horse remains at Cēsis castle, Latvia, and the Teutonic Order's equestrian resources in Livonia. *Med. Archaeol.* **62**, 351–379 (2018).

71. R. A. Bentley, C. Knipper, Geographical patterns in biologically available strontium, carbon and oxygen isotope signatures in prehistoric SW Germany. *Archaeom.* **47**, 629–644 (2005).

72. R. Madgwick, A. Lamb, H. Sloane, A. Nederbragt, U. Albarella, M. Parker Pearson, J. Evans, A veritable confusion: Use and abuse of isotope analysis in archaeology. *Archaeol. J.* **178**, 361–385 (2021).

73. J. A. Evans, C. A. Chenery, K. Mee, C. E. Cartwright, K. A. Lee, A. P. Marchant, L. Hannaford, Data from "Biosphere Isotope Domains GB (V1): Interactive Website." British Geological Survey, https://doi.org/10.5285/3b141dce-76fc-4c54-96fa-c232e98010ea [deposited 1 April 2018].

74. P. Ladegaard-Pedersen, S. Sabatini, R. Frei, K. Kristiansen, K. M. Frei, Testing late bronze age mobility in Southern Sweden in the light of a new multi-proxy strontium isotope baseline of Scania. *PLOS ONE* **16**, e0250279 (2021).

75. J. Moisio, “Beyond the Sea: Migrations and the middle ground in the coastal region of Finland proper during the Roman Iron Age,” thesis, University of Turku, Turku (2015).

76. V. Žulkus, Settlements and Piracy on the Eastern shore of the Baltic sea: The middle ages to modern times. *Archaeol. Balt.* **16**, 58–71 (2011).

77. M. Bertašius, Die Anzeichen einer überregionalen Kultur in den wikingerzeitlichen Gräbern von Marvelė. *Światowit.* **7**, 15–28 (2012).

78. T. Löffelmann, Sr Analyses from only known Scandinavian cremation cemetery in Britain illuminate early Viking journey with horse and dog across the North Sea. *PLOS ONE* **18**, e0280589 (2023).

79. N. Blomkvist, S. Brink, T. Lindkvist, The Kingdom of Sweden, in *Christianization and the Rise of Christian Monarchy: Scandinavia, Central Europe, and Rus’ c. 900–1200*, N. Berend, Ed. (Cambridge Univ. Press, 2007), pp. 167–213.

80. J. H. Lind, Denmark and early Christianity in Finland. *Suomen Museo–Finskt Mus.* **113**, 39–54 (2007).

81. R. Ylimaunu, S. Lakomäki, T. Kallio-Seppä, P. R. Mullins, R. Nurmi, M. Kuorilehto, Borderlands as spaces: Creating third spaces and fractured landscapes in medieval Northern Finland. *J. Soc. Archaeol.* **14**, 244–267 (2014).

82. K. Parppei, Early population in the Ladoga region, in *Lake Ladoga: The Coastal History of the Greatest Lake in Europe*, M. Lähteenmäki, I. Land, Eds. (Finnish Literature Society, 2023), pp. 69–88.

83. I. M. Mulk, T. Bayliss-Smith, Colonisation, Sámi sacred sites and religious syncretism, C. AD 500-1800, in *The Sound of Silence: Indigenous Perspectives on the Historical Archaeology of Colonialism*, T. Äikäs, A. K. Salmi, Eds. (Berghahn, 2019), pp.39–70.

84. J. Scorrer, K. E. Faillace, A. Hildred, A. J. Nederbragt, M. B. Anderson, M.-A. Millet, A. L. Lamb, R. Madgwick, Diversity aboard a Tudor warship: Investigating the origins of the *Mary Rose* crew using multi-isotope analysis. *Roy. Soc. Open Sci.* **8**, 202106 (2021).

85. A. O. Nier, The isotopic constitution of strontium, barium, bismuth, thallium and mercury. *Phys. Rev.* **54**, 275–278 (1938).

86. R. Avanzinelli, E. Boari, S. Conticelli, L. Francalanci, L. Guarnieri, G. Perini, C. Petrone, S. Tommasini, M. Ulivi, High precision Sr, Nd, and Pb isotopic analyses using the new generation thermal ionisation mass spectrometer thermofinnigan triton-Ti®. *Period. di Mineral.* **74**, 147–166 (2005).

87. S. J. Romaniello, M. P. Field, H. B. Smith, G. W. Gordon, M. H. Kim, A. D. Anbar, Fully automated chromatographic purification of Sr and Ca for isotopic analysis. *J. Anal. At. Spectrom* **30**, 1906–1912 (2015).

88. M. B. Andersen, C. H. Stirling, E.-K. Potter, A. N. Halliday, S. G. Blake, M. T. McCulloch, B. F. Ayling, M. J. O'Leary, The timing of sea-level high-stands during Marine Isotope Stages 7.5 and 9: Constraints from the uranium-series dating of fossil corals from Henderson Island. *Geochim. Cosmochim.* **74**, 3598–3620 (2010).

89. B. Wallner, C. Vogl, P. Shukla, J. P. Burgstaller, T. Druml, G. Brem, Identification of genetic variation on the horse Y chromosome and the tracing of male founder lineages in modern breeds. *PLOS ONE* **8**, e60015 (2013).

90. P. M. Grootes, M.-J. Nadeau, A. Rieck, ^{14}C-AMS at the Leibniz-Labor: Radiometric dating and isotope research. *Nucl. Instrum. Methods Phys. Res. B: Beam Interact. Mater. At.* **223**, 55–61 (2004).

91. M. J. Nadeau, P. M. Grootes, M. Schleicher, P. Hasselberg, A. Rieck, M. Bitterling, Sample throughput and data quality at the Leibniz-Labor AMS facility. *Radiocarbon.* **40**, 239–245 (1997).

92. P. J. Reimer, W. E. Ausin, E. Bard, A. Bayliss, P. G. Blackwell, C. B. Ramsey, M. Butzin, H. Cheng, R. L. Edwards, M. Friedrich, P. M. Grootes, The IntCal20 Northern Hemisphere radiocarbon age calibration curve (0-55 cal kBP). *Radiocarbon.* **62**, 725–757 (2020).

93. E. Oras, V. Lang, E. Rannamäe, L. Varul, M. Konsa, J. Limbo-Simovart, G. Vedru, M. Laneman, M. Malve, T. D. Price, Tracing prehistoric migration: Isotope analysis of Bronze and Pre-Roman Iron Age coastal burials in Estonia. *Est. J. Archaeol.* **20**, 3–32 (2016).

94. M. Lahtinen, L. Arppe, G. Nowell, Source of strontium in archaeological mobility studies—Marine diet contribution to the isotopic composition. *Archaeol. Anthropol. Sci.* **13**, 1–10 (2021).

95. E. Petersone-Gordina, J. Montgomery, A. R. Millard, G. Nowell, J. Peterkin, C. A. Roberts, G. Gerhards, V. Zelčs, Strontium isotope identification of possible rural immigrants in 17th century mass graves at St. Gertrude Church cemetery in Riga, Latvia. *Archaeom.* **64**, 1028–1043 (2022).

96. M. Zieliński, J. Dopieralska, S. Królikowska-Ciągło, A. Walczak, Z. Belka. Mapping of spatial variations in Sr isotope signatures ($^{87}Sr/^{86}Sr$) in Poland—Implications of anthropogenic Sr contamination for archaeological provenance and migration research. *Sci. Tot. Environ.* **775**, 145792 (2021).

97. M. Zieliński, J. Dopieralska, Z. Belka, A. Walczak, M. Siepak, M. Jakubowicz, The strontium isotope budget of the Warta River (Poland): Between silicate and carbonate weathering, and anthropogenic pressure. *App. Geochem.* **81**, 1–11 (2017).

98. L. A. Gregoricka, T. K. Betsinger, A. B. Scott, M. Polcyn, Apotropaic practices and the undead: A biogeochemical assessment of deviant burials in post-medieval Poland. *PLOS ONE* **9**, e113564 (2014).

99. M. Larsson, O. Magnell, A. Styring, P. Lagerås, J. Evans, Movement of agricultural products in the Scandinavian Iron Age during the first millennium AD: $^{87}Sr/^{86}Sr$ values of archaeological crops and animals in southern Sweden. *Star* **6**, 96–112 (2020).

100. T. Ahlström, T. D. Price, Mobile or stationary? An analysis of strontium and carbon isotopes from Västerbjers, Gotland, Sweden. *J. Archaeol. Sci. Rep.* **36**, 102903 (2021).

101. T. D. Price, C. Arcini, I. Gustin, L. Drenzel, S. Kalmring, Isotopes and human burials at Viking Age Birka and the Mälaren region, east central Sweden. *J. Anthropol. Archaeol.* **49**, 19–38 (2018).

102. K.-G. Sjögren, T. D. Price, T. Ahlström, Megaliths and mobility in south-western Sweden. Investigating relationships between a local society and its neighbours using strontium isotopes. *J. Anthropol. Archaeol.* **28**, 85–101 (2009).

103. M. Blank, K.-G. Sjögren, C. Knipper, K. M. Frei, J. Storå, Isotope values of the bioavailable

strontium in inland southwestern Sweden—A baseline for mobility studies. *PLOS ONE* **13**, e0204649 (2018).

104. T. D. Price, M. Naum, P. Bennike, N. Lynnerup, K. M. Frei, H. Wagnkilde, T. Pind, F. O. Nielsen, Isotopic investigation of human provenience at the eleventh century cemetery of Ndr. Grødbygård, Bornholm, Denmark. *Dan. J. Archaeol.* **1**, 93–112 (2012).

105. H. Wilhelmson. T. D. Price, Migration and integration on the Baltic island of Öland in the Iron Age. *J. Archaeol. Sci. Rep.* **12**, 183–196 (2017).
